# Changes in starch structures and in vitro digestion characteristics during maize (*Zea mays* L.) germination

**DOI:** 10.1002/fsn3.1457

**Published:** 2020-02-13

**Authors:** Xianhong Ma, Yang Liu, Jingsheng Liu, Jingjing Zhang, Renning Liu

**Affiliations:** ^1^ College of Food Science and Engineering Jilin Agricultural University Changchun China; ^2^ College of Biotechnology and Food Engineering Jilin Institute of Chemical Technology Jilin China; ^3^ National Engineering Laboratory of the Wheat‐corn Deep Processing Changchun China

**Keywords:** digestion, germination, maize starch, structures

## Abstract

This study analyzed changes in the starch structures and in vitro digestion profiles of a specific maize cultivar, Jike 728 (JK728), in Jilin, China, after 0–5 days of germination. The total starch, amylose, and amylopectin contents decreased significantly during germination. The average molecular weight of the starch compounds also decreased significantly during germination. The proportion of amylopectin with a degree of polymerization (DP) of 13–24 significantly decreased, while the relative abundance of amylopectin with DP values of 6–12, 25–36, and 37–60 significantly increased. The X‐ray diffraction (XRD) patterns of all samples were characteristic of A‐type starch, and the starch relative crystallinity decreased over time. The proportions of slowly digestible starch and resistant starch decreased significantly, while the proportion of rapidly digestible starch increased significantly during germination. Germination is an easy, inexpensive, and low‐carbon processing method. This study indicates that germination is an effective way to control the physical properties and digestibility of starch in maize. The changes observed in the physical properties and digestibility of maize starch after germination provide scientists with a platform to understand starch modification mechanisms that might have potential applications on an industrial scale.

## Graphical abstract

The total starch, amylose, and amylopectin concentrations decreased significantly during maize germination (0–5 days). The proportions of amylopectin with a DP of 13–24 significantly declined and those with a DP of 6–12, DP of 25–36, and DP of 37–60 significantly rose during maize germination (0–5 days). The XRD patterns of all starch samples were characteristic of A‐type starch, and the starch relative crystallinity was reduced during maize germination (0–5 days). The SDS and RS levels significantly declined, and the RDS levels significantly rose during maize germination (0–5 days).

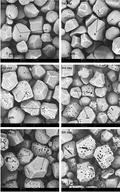

## 1 INTRODUCTION

The corn plant maize (*Zea mays* L.) is a member of the Gramineae family. Maize is rich in nutrients and is composed of 8% protein, 0.8% fat, 79.2% carbohydrates, and 14.4% dietary fiber. Maize is also rich in vitamins and mineral elements. Furthermore, maize is an important feed source in the animal husbandry and aquaculture sectors and is an indispensable raw material in relation to food, healthcare, and chemical industries. There is a high starch content (70%) in maize. Thus, maize starch plays an important role in the maize processing industry.

Starch is the main carbohydrate in the human diet and serves as the main source of human energy. This polysaccharide can be categorized into three types based upon the characteristics of digestion: rapidly digestible starch (RDS), slowly digestible starch (SDS), and resistant starch (RS) (Englyst, Kingman, & Cummings, 1992). Among these types, RDS can be rapidly digested in the small intestine, whereas slowly digestible starch SDS can be slowly and fully digested in the small intestine. By contrast, RS cannot be digested in the small intestine of healthy people but, together with its degradation products, can be fully or partially fermented in the large intestine.

During germination, vital kernels activate and synthesize endogenous enzymes after water absorption and a requisite period of respiration. These enzymes subsequently promote the exit of the kernel capsules and the exposition of the kernel roots from the kernel embryos. Following the activity of the endogenous enzymes during germination, macromolecules (e.g., proteins, fats, carbohydrates) are decomposed and nutrients (e.g., vitamins, minerals) are released, thereby causing changes to kernel composition (Lee et al., 2018; Sangsukiam & Duangmal, 2017). The starch content in oats after 6 days of germination has been shown to significantly decrease from 60% to 20% (Tian et al., 2010). During the germination of three cultivars of brown rice (0–5 days), total starch, amylose, and amylopectin contents all significantly decreased (Wu et al., 2013). Moreover, the effects of germination on starch digestion were studied in vitro. Xu, Zhang, Guo, and Qian (2011) tested the effects of germination on brown rice starch digestion in vitro and found that the SDS level increased by 10.09% after 24 hr; however, the RDS and RS levels decreased by 11.95% and 11.71%, respectively. After 36 hr of germination, the RDS level in glutinous brown rice increased by 18.5%, while the SDS and RS levels dropped by 14.5% and 4.0%, respectively (You et al., 2016). The results of the aforementioned studies imply that germination moderately affects grain starch digestion.

Maize is one of the most common cereal grains and yields for this crop rank only after rice and wheat. Since the content of starch in maize is approximately 70%, maize represents an important source of starch. However, there are many limitations pertaining to natural starch that affect its application. As a result, many researchers have used chemical (Chhabra, Kaur, & Kaur, 2018), physical (Bahrani et al., 2017; Kaur & Singh, 2018), biological (Bird, Brown, & Topping, 2000), and enzymatic methods (Li et al., 2019; Zhang et al., 2017) to modify the structure of starch and expand the application scope of this biomaterial. Chemical modification is a relatively mature method of starch modification; however, the major drawback in relation to this strategy involves the presence of solvent residues. Some countries have banned or restricted the application of chemically modified starch in food (Singh, Inouchi, & Nishinari, 2006). The most commonly used methods of physical modification include high‐pressure treatment, hydrothermal treatment, and the addition of compounds. Although physically modified starch does not contain solvent residues, there is an increased requirement for expensive equipment to facilitate this strategy. Biological methods improve starch properties by altering the genes of target enzymes in starch biosynthesis pathways. However, biological methods have very specific requirements and often require customized strategies, which do not often improve the properties of starch. Enzymatic methods are mild, efficient, and environmentally friendly. Germination is often employed as a starch modification strategy, and associated methodologies require the use of endogenous enzymes. Thus far, the effects of germination on the structures and digestibility of maize starch have been rarely studied. Teli, Rohera, Sheikh, and Singhal (2009) studied the application of maize starch germinated by moisture in printing during poor storage conditions. However, the latter study did not systematically study the effect of germination on the structure of maize starch. Thus, this study aimed to clarify the effects of germination on the structures and in vitro digestibility of maize starch, thereby offering an additional reference in relation to the modification and application of maize starch. For this purpose, we selected the common maize cultivar, JK728, for investigation. We systematically explored the total starch, amylose, and amylopectin contents, the morphological changes in the starch granules, the molecular weight of starch, the distribution of amylopectin branch chains (fraction), the X‐ray diffraction (XRD) patterns, starch relative crystallinity, and the changes in RDS, SDS, and RS contents during 0–5 days of maize germination.

## 2 MATERIALS AND METHODS

### 2.1 Materials

Jike 728 (jk728), a common maize in Northeast China, was harvested in Jinzhou, Liaoyuan, China (43^。^03' N, 125^。^17' E). Maize sticks were collected manually when the moisture content was 35% ± 3%, and the maize seeds were subsequently stripped (manually). The resultant maize was stored at a temperature of 20 ± 2°C and a relative humidity of 55% ± 5%. These conditions ensured that there was no invasion of insects and mold during storage.

### 2.2 Preparation of germinated maize

Methods published by Wu, Yang, Chen, Jin, and Xu (2011) were utilized in this study with some minor modifications. The maize kernels were screened to remove nonplump specimens; then, 4,000 g of kernels were soaked in a 0.1% NaClO solution (8 L) for 30 min and rinsed with deionized water. Next, the maize kernels were laid flat on a two‐layer multifunctional sprout dish, covered by wet two‐layer gauze, and deionized water (1 cm high) was added to the lower layer. Then, the dish was placed into an incubator to facilitate germination at a constant temperature (25°C) and humidity level. The maize kernels were rinsed with deionized water for 1 min at 6 hr intervals and then sampled at 24 hr intervals. The kernels were subsequently prepared and then dried in a desiccator at 40°C after 0, 1, 2, 3, 4, and 5 days of germination until a water content of 15% ± 2% was reached. Finally, the maize kernels were stored in sealed plastic bags, which were placed in a refrigerator at 4°C until further required.

### 2.3 Starch extraction

A method published (with minor modifications) by Lin and Chang (2006) was utilized to facilitate starch extraction. Firstly, 100 g of the dried germinated maize was weighed and added to 1,000 ml of 0.2% NaHSO_3_ solution. After soaking at 40°C for 50 hr, the maize was washed 3 times with deionized water and cleaned by manually removing the cortex, root cap, and germ. The resultant endosperm was added into 1,000 ml of deionized water and ground at 4,000 *g* for 1 min on an 878‐A multifunctional tissue crusher (Guohua Instruments Co. Ltd). The resultant liquids were subsequently centrifuged in an Allegra X‐30R high‐speed freezing centrifuge (Beckman Coulter, Inc.) at 4,000 *g* for 10 min. The supernatant was discarded, the upper layer of soft yellow starch was scraped off, and the precipitates were washed three times with deionized water. Next, the centrifugation step was repeated, the supernatant was discarded, and the soft yellow starch was removed. The lower layer of starch was oven‐dried at 40°C for 24 hr, crushed, and filtered (100 mesh). The resultant starch powder was stored in a dryer until further required.

### 2.4 Measurement of total starch, amylose, and amylopectin contents

The total starch contents of the maize flour were measured using an enzymatic method (McCleary, Gibson, Solah, & Mugford, 1994) (with minor modifications). Each maize flour sample (100 mg) was placed into a 15 ml test tube. Next, 0.2 ml of 85% ethanol solution followed by 3 ml of 0.5% α‐amylase (E.C3.2.1.1; Sigma) were added to the tubes that were subsequently incubated at 95°C for 6 min with intermittent mixing. Finally, 0.1 ml of amyloglucosidase (A7095; Sigma) was added to each tube and incubated at 50°C for 30 min. Next, the liquid in each test tube was transferred to a 100 ml volumetric flask and the associated volumes were fixed. The supernatant (0.1 ml) was then added to the test tube. Three milliliters of glucose oxidase (D‐glucose assay kit; Megazyme) were subsequently added and the mixture was incubated at 50°C for 20 min. Next, 0.1 ml of the glucose standard solution and 3 ml of glucose oxidase (D‐glucose assay kit, Megazyme) reagent were added and the mixtures were incubated at 50°C for 20 min. Water (0.1 ml) was added to the reagent blank. The absorbance was measured at 510 nm. Total starch contents were measured according to an absorbance‐based method proposed by McCleary et al. (1994). The amylose contents were detected by the iodometric method (McGrance, Cornell, & Rix, 1998) (with minor modifications). Ten milligrams of each sample were subsequently weighed into clean EP tubes, and 100 µl of 85% ethanol and 900 µl of 2 mol/L NaOH solution were added to each sample. Each sample was subsequently vortexed, mixed well, and boiled for 10 min. The samples were then allowed to cool. Next, 0.5 ml of supernatant, 0.1 ml of 2 mol/L acetic acid, and 0.2 of ml potassium iodide solution were added to each tube, and the tubes were left at room temperature for 10 min. The absorbance was measured at 720 nm. The amylopectin content was determined as the total starch content minus the amylose content.

### 2.5 Scanning electron microscopy (*SEM*) of granule morphology

In accordance with a method published by You et al. (2016) (with minor modifications), the starch powder was fixed via a conductive double‐sided adhesive onto a sample platform and, after sputter coating with gold, was placed under a Phenom ProX *SEM* device (Phenom‐World B.V.) for observation with a 10 kV electron beam. The morphological changes in the starch granules were observed, and representative photographs were taken.

### 2.6 Measurement of starch molecular weights

In accordance with a previously reported method (Wu et al., 2013) with minor modifications, 20 mg of starch was weighed and dissolved in 10 ml of a 90% dimethyl sulfoxide (DMSO) solution containing 50 mmol/L LiBr. The resultant solution was heat‐stirred on a magnetic stir plate at 100°C for 1 hr. Next, the temperature was adjusted to 24°C, and the solution was further stirred for 24 hr. After 15 min of centrifugation at 3,000 *g*, the supernatant was filtered through a 0.45 µm microporous film and injected into a Waters1525 high‐performance liquid‐phase size‐exclusion chromatography (Waters)—Dawn DSP‐F multiangle laser scattering meter (Wyatt Technology Co.)—Optilab T‐rEx differential refractive index detector (Wyatt Technology Co.) system (HPSEC‐MALLS‐RI). The chromatogram conditions were as follows: mobile phase was 90% DMSO solution containing 50 mmol/L LiBr, flow velocity was 0.5 ml/min, and column temperature was 60°C.

### 2.7 Analysis of amylopectin branch chains (fraction)

The **amylopectin branch chain (fraction)** distribution was analyzed using high‐performance size‐exclusion chromatography (HPSEC) (e2695, Waters corp., Chicago, USA) coupled to a refractive index detection (HPSEC‐RI) system. In accordance with a previously reported method (You et al., 2016) with minor modifications, 10 mg of starch was dispersed in 2 ml of a 90% DMSO solution. This mixture was subsequently treated in a boiling water bath for 20 min. After cooling, the solution was uniformly mixed with 6 ml of methanol and then centrifuged at 3,000 *g* for 10 min. The precipitates were dissolved in 2 ml of sodium acetate buffer (50 mM, pH 3.5) and then treated in a boiling water bath for 20 min. After cooling to 37°C, 5 µl of isoamylase (1,000 U/µl, I5284; Sigma) was added, and the mixture was incubated at 37°C for 24 hr. Isoamylase was subsequently inactivated in a boiling water bath for 10 min. After cooling to 4°C, the resultant solution was centrifuged at 3,000 *g* for 10 min. Next, the supernatant was degassed and filtered through a 0.22 μm cellulose ester microporous membrane. The supernatant was subsequently analyzed by HPSEC‐RI. The chromatogram conditions were as follows: A Shodex SB‐803 (1,000–100,000) column (Shodex) and a Shodex SB‐802.5 (300–10,000) column (Shodex) were connected according to the separation ranges from large to small. A Shodex SB‐G (Shodex) protection column was connected in front of the analytical column. The column temperature was set at 40°C. The mobile phase was 0.1 mol/L Tris, 0.1 mol/L NaCl, and pH 7.40, and the flow rate was 0.5 ml/min. The Shodex RI‐101 differential refractive index detector (Shodex) was set at 37°C. Samples were fed via a trace flat injector at a concentration of 10 g/L and dosage of 20 µl. The normalization analysis and peak area calculation were carried out on Origin 9.0 (Microcal). The peak area ratio of the amylopectin branch chain (fraction) is its DP.

### 2.8 X‐ray diffraction (XRD) and relative crystallinity

The relative crystallinity of the maize starch was detected on a D8 Avance XRD meter (Bruker Corp.) using CuKa rays, a tube voltage of 35 kV, a tube ampage of 20 mA, a scanning range of 5–40°, and a scanning rate of 0.02°/s using a method previously published by Zhang et al. (2017) with minor modifications. Origin 9.0 software (Microcal) was used to calculate the relative crystallinity of maize starch according to the two‐phase method described by Lopez Rubio, Flanagan, Gilbert, and Gidley (2008).

### 2.9 Analysis of starch following in vitro digestion

Starch digestibility was measured using an in vitro simulated enzyme hydrolysis method (Englyst et al., 1992) with modifications. First, an enzyme mixture was prepared: 3.0 g of porcine pancreas α‐amylase (P7545; Sigma) was placed into a 50‐ml centrifuge tube. Ten milliliters of 0.1 mol/L sodium acetate buffer were subsequently added, and the mixture was subjected to magnetic stirring. The mixture was next centrifuged at 1,500 *g* for 10 min. The supernatant was then added to 1.0 ml of amyloglucosidase (A7095; Sigma).

Then, 0.3 g of starch was placed into a screw‐cap centrifuge tube. Next, 7.5 ml of 0.1 mol/L sodium acetate buffer (pH 5.2) was added, and the mixture was placed into a water bath at 95°C for 30 min to facilitate gelatinization. Five glass beads and 0.75 ml of the enzyme mixture were added, and the mixture was shaken at 200 *g* in a water bath at 37°C. After 20 and 120 min, 0.5 ml of the supernatant was collected and added to 20 ml of 95% ethanol to denature the enzymes. The resultant solutions were centrifuged at 1,500 *g* for 10 min. Next, the glucose contents in the starch solutions after 0, 20, and 120 min of hydrolysis were detected via a glucose oxidase method (d‐glucose assay kit; Megazyme). The starch contents were calculated as follows:

$$\text{RDS}=\frac{(G_{20}-G_{0})\times 0.9}{\text{TS}}\times 100\%$$

$$\text{SDS}=\frac{(G_{120}-G_{20})\times 0.9}{\text{TS}}\times 100\%$$

$$\text{RS}=\frac{\text{TS}-(\text{RDS}+\text{SDS})}{\text{TS}}\times 100\%$$

where G20 is the glucose content after 20 min of amylase hydrolysis (mg),G0 is the free glucose content before enzyme hydrolysis (mg),G120 is the glucose content after 120 min of amylase hydrolysis (mg), and.TS is the total starch (mg).

### 2.10 Statistical analysis

The experiments were repeated at least three times, and the results were expressed in the form of mean ± standard deviation. The statistical software SPSS 19.0 (SPSS 19.0 for Windows, SPSS Inc) was used to analyze the experimental data, and Duncan's Multiple Range test in ANOVA was used to analyze the difference (*p* < .05).

## 3 RESULTS AND DISCUSSION

### 3.1 Changes in total starch, amylose, and amylopectin contents during maize germination

Changes to the starch composition during maize germination are listed in Table 1. The total starch, amylose, and amylopectin contents all significantly decreased as the germination of the maize progressed. The associated rates gradually increased during the first three days but then decreased for each of the aforementioned parameters. These results are consistent with those of a previous study published by Wu et al., (2013). The starch content decreased during germination as starches were converted into low‐molecular‐weight sugars to meet associated energy requirements (Tian et al., 2010).

**Table 1:** Changes in total starch, amylose, and amylopectin concentrations, and relative crystallinity during maize germination (0–5 days)

Days of germination	Total starch (g∙100 g^−1^)	Amylose (g∙100 g^−1^)	Amylopectin (g∙100 g^−1^)	Relative crystallinity (%)
0	69.21 ± 0.17^a^	15.53 ± 0.08^a^	53.68 ± 0.19^a^	23.53 ± 0.15^a^
1	64.77 ± 0.21^b^	14.36 ± 0.54^b^	50.42 ± 0.74^b^	21.86 ± 0.19^b^
2	57.35 ± 0.10^c^	12.52 ± 0.47^c^	44.83 ± 0.52^c^	21.43 ± 0.18^b^
3	47.45 ± 0.19d	9.91 ± 0.46^d^	37.54 ± 0.57^d^	21.08 ± 0.06^c^
4	41.88 ± 0.25^e^	8.53 ± 0.36^e^	33.35 ± 0.51^e^	20.53 ± 0.02^d^
5	36.34 ± 0.10^f^	7.22 ± 0.37^f^	29.12 ± 0.40^f^	15.22 ± 0.07^e^

Note

The experimental data were measured more than three times, and the results are expressed in the form of mean ± standard deviation. Significant differences in the data in the same index are expressed by different lowercase letters (*p < *.05).

### 3.2 Starch granule morphology of germinated maize observed via *SEM*

**Figure 1:**
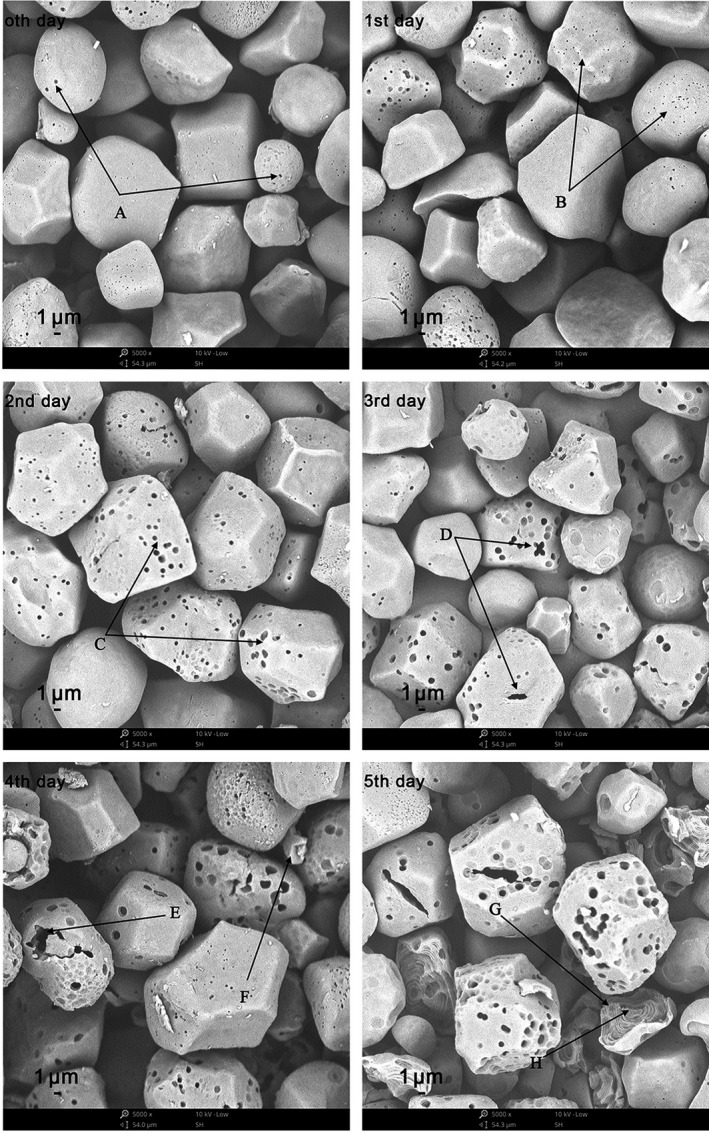
*SEM* images of maize starch during germination (0–5 days) (magnification values ×5,000). A/B/C/D/E: Holes; F: fragments; G: wheel veins; H: kernel center

Figure 1 shows the *SEM* images of maize starch during germination. The native maize starch and germinated maize starch granules are both irregular polygons. The images on day 0 show that the raw maize starch granules contained pore‐like structures (A), or "holes." These structures are also found in broomcorn starch, millet starch, wheat starch, and barley starch (Huber & BeMiller, 2000). The pores in the starch can be generated during either granule formation or the starch isolation process (Jane et al., 2003). The image on the first day of germination was not largely different from the images on day 0 (B). As the germination time progressed, the maize starch granules obviously changed. On the second day, the holes were slightly enlarged (C). On the third day, several holes connected to form larger holes (D). On the fourth day, the holes were further enlarged (E), forming kernel fragments (F). On the fifth day, starch granule layers were observed, and dark‐bright alternative onion‐like ring‐shape structures were formed. In some areas, ring veins and wheel veins were apparent (G). All of the aforementioned rings encircled the kernel center (H). These wheel‐shaped structures were considered growth rings of starch granules and were formed between the crystal and amorphous layers (Jane, Kasemsuwan, Leas, Zobel, & Robyt, 1994).

### 3.3 Effects of germination on molecular weights of maize starch

The molecular weights of maize starch during germination are shown in Table 2. As shown, the weight‐average molecular weights (*M*_w_) and number‐average molecular weights (*M*_n_) of maize starch both decreased significantly during germination. These results are consistent with those published by Xu et al. (2011) and You et al. (2016). This phenomenon may be due to the molecular degradation of starch during maize germination, resulting in a decrease in the *M*_w_ and *M*_n_. During germination, high‐molecular‐weight sugars were degraded into low‐molecular‐weight sugars, in order to generate the requisite energy required for germination (Yiming et al., 2015). Hence, the degradation of starch into low‐molecular‐weight sugars led to a decrease in *M*_w_ and *M*_n_. It was previously reported that the *M*_w_/*M*_n_ ratio increased after germination (Wu et al., 2013; Xu et al., 2011). Moreover, the starch dispersity (*M*_w_/*M*_n_) increased from 2.20 ± 0.16 to 3.14 ± 0.36 after germination, indicating that the starch granules became nonuniform after germination.

**Table 2:** Changes in starch molecular weights during germination (0–5 days)

Parameter	Days of germination					
	0	1	2	3	4	5
*M*_w_ (×10^7^ g^.^mol^−1^)	3.37 ± 0.24^a^	3.06 ± 0.21^a^	2.60 ± 0.22^b^	2.07 ± 0.26^c^	1.79 ± 0.2^cd^	1.46 ± 0.35^d^
*M*_n_ (×10^7^ g^.^mol^−1^)	1.54 ± 0.23^a^	1.30 ± 0.22^ab^	1.04 ± 0.18^bc^	0.73 ± 0.21^cd^	0.60 ± 0.13^d^	0.46 ± 0.06^d^
*M*_w_/*M*_n_	2.20 ± 0.16^c^	2.39 ± 0.33^bc^	2.54 ± 0.26^abc^	2.91 ± 0.48^ab^	3.03 ± 0.41^ab^	3.14 ± 0.36^a^

Note

The experimental data were measured more than three times, and the results were expressed in the form of mean ± standard deviation. The significantly different data in the same index are expressed by different lowercase letters (*p* < .05). The weight‐average molecular weights are expressed in *M*_w_, and the number‐average molecular weights is expressed in *M*_n_.

### 3.4 Effects of germination on the amylopectin branch chain (fraction)

Table 3 illustrates the effects of germination on the amylopectin branch chain (fraction). Specifically, the amylopectin branch chain (fraction) distributions differed significantly with germination time. Hanashiro, Abe, and Hizukuri (1996) categorized amylopectin branch chain (fraction) into four types: A chains were DP (6–12), B1 chains were DP (13–24), B2 chains were DP (25–36), and B3 chains were DP ≥37. The proportion of the maize amylopectin branch chain segment on DP (13–24) significantly decreased with prolonged germination. The proportions of three maize amylopectin branch chain segments on DP (6–12), DP (25–36), and DP (37–60) significantly increased with prolonged germination. During maize germination, branch types A and B of amylopectin were degraded into oligosaccharides (e.g., maltose, maltotriose, linear dextrin). This latter phenomenon led to an increase in the fraction of short‐chain DP (6–12) (Angellier‐Coussy et al., 2009; Bertoft, 2013). In this study, the HPSEC‐RI system used to detect amylopectin branch chain (fraction) distributions did not facilitate the detection of super‐long amylopectin branch chain (fraction) above DP 60. Thus, it can be hypothesized that the increasing proportions of long amylopectin branch chain (fraction) that were observed were caused by the presence of undetected super‐long amylopectin branch chains (fraction) (Hizukuri, 1985; You et al., 2016).

**Table 3:** Changes in amylopectin branch chain (fraction) during germination (0–5 days)

Parameter	Days of germination					
DP (%)	0	1	2	3	4	5
6 ~ 12	33.21 ± 0.12^f^	34.44 ± 0.50^e^	35.15 ± 0.18^d^	37.41 ± 0.51^c^	38.38 ± 0.15^b^	39.13 ± 0.07^a^
13 ~ 24	45.8 ± 0.60^a^	41.60 ± 0.55^b^	38.27 ± 0.25^c^	31.35 ± 0.22^d^	28.24 ± 0.45^e^	25.88 ± 0.45^f^
25 ~ 36	12.35 ± 0.48^f^	13.58 ± 0.06^e^	14.28 ± 0.03^d^	16.54 ± 0.49^c^	17.81 ± 0.13^b^	18.57 ± 0.33^a^
37 ~ 60	8.63 ± 0.13^f^	10.38 ± 0.30^e^	12.30 ± 0.11^d^	14.71 ± 0.22^c^	15.58 ± 0.34^b^	16.42 ± 0.29^a^

Note

The experimental data were measured more than three times, and the results are expressed in the form of mean ± standard deviation. The significantly different data in the same index are expressed by different lowercase letters (*p* < .05).

### 3.5 X‐ray diffraction and relative crystallinity (XRD)

**Figure 2:**
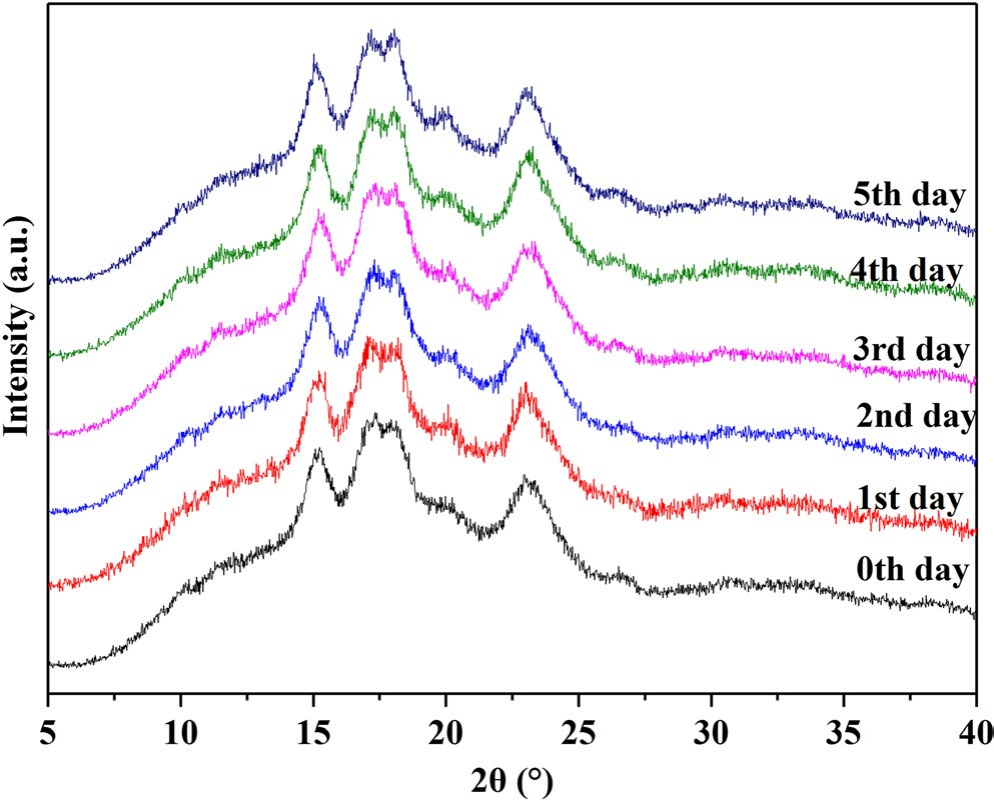
XRD patterns of maize starch after 0–5 days of germination

JK728 exhibited strong diffraction peaks at 15.550, 17.510, 18.450, and 23.646 before germination (Figure 2). The XRD patterns indicate that the maize starch belongs to A‐type starch (Oates, 1997). Following 1–5 days of germination, apart from the fact that the peak positions changed (they still belonged to A‐type starch), the original characteristic peaks were preserved in the maize starch. As shown in Table 1, the relative crystallinity was reduced over time for the maize starch. This finding is consistent with results published by Teli et al. (2009), which showed that the relative crystallinity of maize decreases during germination. Cheetham and Tao (1998) suggested that the differences in the amylopectin branch chain (fraction) might result in differences in the relative crystallinity of amylopectin. Gidley and Bulpin (1987) reported that the increase in the proportion of short amylopectin branch chain (fraction) (DP <10) may lead to a decrease in the stability of the double helix structure of amylopectin. In this study, the relative abundance of short‐chain DP (6–12) in amylopectin increased significantly with germination time. An increase in the fraction of short‐chain DP (6–8) may account for the decrease in crystallinity, since short‐chain DP (6–8) is too short to partake in crystal formation and only chains with DP >8 can form crystals.

### 3.6 Changes in in vitro digestion during germination

**Figure 3:**
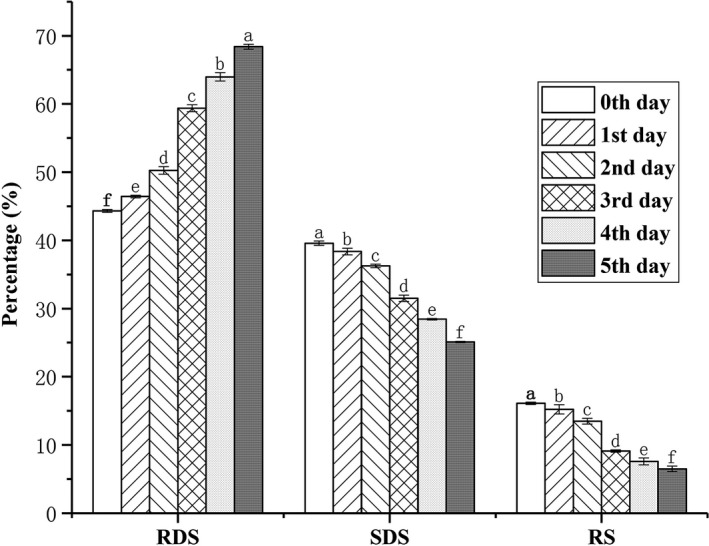
In vitro changes in starch digestion during germination (0–5 days). The significantly different data in the same index are expressed by different lowercase letters (*p* < .05). The rapidly digestible starch is abbreviated as RDS, the slowly digestible starch is abbreviated as SDS, and the resistant starch is abbreviated as RS

Changes to starch following in vitro digestion during maize germination are listed in Figure 3. The RDS contents significantly increased, whereas the SDS and RS contents both significantly decreased with prolonged germination time. This latter result indicates that germination significantly affects maize starch digestion in vitro. Frias, Fornal, Ring, and Vidal‐Valverde (1998) reported an increase in the digestibility of starch after germination of lentils. Chung, Cho, Park, Kweon, and Lim (2012) reported that the digestibility of starch after germination of brown rice also increased. The authors of the latter study speculated that the increase in starch digestibility during germination may be due to degradation of the starch by amylolytic enzymes. This phenomenon results in the easy digestion of the starch. Chung, Moon, Kim, and Chun (2002) and Hoover (2001) believed that factors including particle morphology, molecular structure, and relative crystallinity of amylopectin affect starch digestibility. In the present study, *SEM* (Figure 1) analysis revealed that the pores on the surface of starch granules became larger with germination time. They also reported that starch granule layers appeared as germination progressed. This phenomenon may improve the penetration efficiency of the enzyme in the starch granules. As the germination progressed, the relative crystallinity of the starch decreased significantly (Table 1), a factor that might also accelerate the degradation of the starch. The proportion of short‐chain DP (6–12) in amylopectin increased significantly with germination time (Table 3). As the germination progressed, the molecular weight of maize starch decreased significantly (Table 2), indicating partial degradation of the starch, which may have resulted in the increased digestibility.

## 4 CONCLUSIONS

During 0–5 days of germination, the relative abundance of amylopectin branch chain segments with a DP of 13–24 significantly decreased, whereas the relative abundance of amylopectin branch chain segments with a DP of 6–12, 25–36, and 37–60 significantly increased over time. The XRD patterns of all the starch samples assessed are characteristic of A‐type starch, and the relative crystallinity was reduced with time. The in vitro digestion analysis showed that the SDS and RS levels both significantly decreased, while the RDS levels significantly increased. This study indicates that germination is an effective way to improve the RDS content of maize starches.

## CONFLICT OF INTEREST

The authors declare no conflict of interest.

## ETHICAL STATEMENT

This study does not involve any human or mammalian testing.
